# Dissemination and Implementation Approach to Increasing Access to Local Pre-Exposure Prophylaxis (PrEP) Resources With Black Cisgender Women: Intervention Study With Vlogs Shared on Social Media

**DOI:** 10.2196/67367

**Published:** 2025-03-28

**Authors:** Mandy J Hill, Laurenia Mangum, Sandra J Coker, Tristen Sutton, Diane M Santa Maria

**Affiliations:** 1 Department of Emergency Medicine, McGovern Medical School, UTHealth Houston, 6431 Fannin St, Houston, TX, 77030, United States, 1 713-500-7661; 2 Jane Addams College of Social Work, University of Illinois Chicago, Chicago, IL, United States; 3 Tristen Sutton Consulting, Houston, TX, United States; 4 Cizik School of Nursing, UTHealth Houston, Houston, TX, United States

**Keywords:** PrEP, cisgender Black women, social media campaign, PrEP access, HIV prevention, vlogging, dissemination and implementation, pre-exposure prophylaxis, dissemination, implementation, HIV, prevention, human immunodeficiency virus, cisgender, social media, marketing, campaign, education, sexually transmitted diseases, STDs, vlog

## Abstract

**Background:**

Black cisgender women account for only 2% of pre-exposure prophylaxis (PrEP)-eligible people in the United States who use PrEP to prevent HIV. Owing to the low PrEP use, Black cisgender women continue to contract HIV more frequently than women from every other racial group. Intervention efforts that can bridge the link between knowing that PrEP prevents HIV and support with access to PrEP are necessary for Black cisgender women.

**Objective:**

The purposes of the vlogs through the campaign were to share information about ways to prevent HIV using PrEP and fact-based education and provide access to PrEP resources with active links to local PrEP providers at local community health centers.

**Methods:**

In Phase 1, the study team formerly piloted full-length video blog posts (ie, vlogs; 10‐12 min each) with 26 women during an emergency department visit. Using the findings from Phase 1, Phase 2 involved a prospective 6-month social media marketing campaign. The study team led a Texas-Development CFAR-funded pilot grant to disseminate brief vlog snippets (30 s) of excerpts from full-length vlogs with a larger group of Black women in Harris County. Community members, who were aged 18‐55 years, usually consume content that is often viewed by Black cisgender women (ie, health and beauty) and reside in neighborhoods (based on zip code) in Harris County where most residents are Black or African American. They were shown a series of brief vlog snippets on their social media pages, along with a brief message about PrEP and an active hyperlink to local PrEP resources. The study team assessed implementation outcomes, including the feasibility and acceptability, appropriateness of vlogs, access to PrEP resources at local clinics, and clinical outcomes such as increased PrEP awareness among Black cisgender women.

**Results:**

Within 6 months, the campaign reached 110.8k unique individuals (the number of unique accounts that have seen your content at least once) who identified as women. When stratified by age, video plays (the number of times a video starts playing) at 50% of the vlogs (n=30,877) were most common among women aged 18‐24 years (n=12,017) and least common among women aged 45‐54 years (n=658). Key performance indicators showed that 1,098,629 impressions (the number of times a user saw the vlog) and 1,002,244 total video plays resulted in 15,952 link clicks to local PrEP resources.

**Conclusions:**

The campaign demonstrated the feasibility and acceptability of this approach with Black cisgender women and illustrated preliminary effectiveness at supporting access to local PrEP resources with Black cisgender women. Further dissemination and implementation of this approach is necessary to fully assess whether vlog viewership and clicks on links to PrEP resources can meaningfully empower Black cisgender women to access PrEP and help them to assess whether PrEP is personally a useful HIV prevention option.

## Introduction

### Background

Pre-exposure prophylaxis (PrEP) describes a medication that is proven to be 99% effective when taken as prescribed to reduce the risk of getting HIV by taking a pill or injection among people who do not have HIV but may be vulnerable to being introduced to or “exposed” to it. The marginal use of PrEP hinders progress toward decreasing the incidence of HIV cases among Black cisgender women. Black women account for nearly 60% of new HIV cases among women in the United States while comprising less than 15% of the female population [1,2] and also accounting for 67% of new HIV diagnoses among all women in the southern part of the United States [3]. Additionally, Black women in Houston are 18.4 times more likely to be living with HIV [4] than other women in the United States. Optimal progress toward ending the HIV epidemic (EHE) goals requires strategies that will interrupt transmission pathways among women in HIV hotspot locations. PrEP, when taken as prescribed as an oral daily or bimonthly injection medication, can reduce the risk of HIV through sex by 99% [5,6]. Interim findings of the PURPOSE 1 trial presented at the AIDS 2024 Conference confirm that lenacapavir, a long-acting injectable PrEP technology that can be taken twice a year, offered 100% protection against HIV transmission among cisgender women enrolled [7]. However, less than 2% of eligible Black cisgender women in the United States take PrEP [8].

The critical gap between the availability of PrEP and use by Black cisgender women, arguably the most HIV-vulnerable demographic of women in the United States, requires the attention of researchers and interventionists. The identified barriers to PrEP uptake with Black women include limited PrEP awareness, low perceived HIV risk, concerns about adverse effects, concerns about costs, limited marketing, and distrust in the health care system [9]. Real-life competing priorities (ie, housing instability and food insecurity) trump HIV prevention for many Black women [10]. Social and structural factors, including racism, sexism, and classism, are often barriers to PrEP access, especially among young Black women [10,11]. Black women have cited medical racism as a key reason for distrusting the medical system and, as a result, not choosing to use PrEP [10-13]. These barriers, in addition to legacy of medical mistrust [13-15], the dehumanization of Black women [16], and stigmatizing experiences within health care settings [15-18], create a seemingly impermeable barrier to the possibility of increasing PrEP use among Black women. However, public messaging of PrEP as an effective HIV prevention message via innovation by culturally immersed thought leaders, researchers, and interventionists who are of the community can be believed to be true, real, and relatable for Black cisgender women.

To date, there is a paucity of research focused on behavioral interventions that can overcome the complex barriers that suppress PrEP uptake among Black cisgender women. As such, progress towards EHE has been slow within and among sexual networks of Black cisgender women and there has been no significant decrease in HIV incidence or any meaningful increase in PrEP uptake within the last decade [8], which is a significant unmet population health need.

The time that it takes between intervention development and integration of innovative, evidence-based interventions (EBIs) into routine clinical practice can worsen the structural and systemic barriers that impede PrEP use among Black cisgender women. This process can take over a decade, leaving the science outdated, EBIs being ineffective, and improvement in clinical outcomes unaddressed. The measures of acceptability and feasibility in this study are poised to inform the adoption, scale-up, and sustainability of EBIs intending to increase PrEP initiation from the perspective of Black cisgender women. The acceptability of EBIs typically refers to the perception among stakeholders that a given treatment, service, practice, or innovation is agreeable, or palatable, which can change over time [19]. In this research, we are interested in Black cisgender women’s acceptability of HIV prevention vlogs as a pathway for both information dissemination and linkage to PrEP resources at local clinics. The research team designed the vlogs with an intentional focus on race, gender, and culture to meet the needs of Black cisgender women. Local clinics already use webpages to provide information about PrEP to community members. In this case, vlogs could drive traffic to the clinic webpages. Lastly, the feasibility measure will indicate how well vlogs can be used to motivate access to PrEP. Findings of feasibility and acceptability measures related to the influence of vlog use, as a health communication strategy, through a social media campaign on information access to PrEP knowledge and access to PrEP will produce a meaningful addition to the paucity of research on behavioral interventions that overcome complex barriers to PrEP access and use with Black cisgender women, and may inform pathways to facilitate PrEP uptake with Black women in the future.

Some progress has been made with identifying facilitators to PrEP uptake with Black women, including women’s empowerment and advocacy, a need for increased PrEP-specific education, and the positive influence of PrEP-engaged women’s testimonials [9]. In a study (2017‐2019) where 26% of cisgender women (145/565) in NYC initiated PrEP, Latina women (29.7%, 79/565) and Black women (26.1%, 47/565) were more likely to initiate PrEP when compared to White women (16.3%, 14/565) [10]. Findings demonstrated that PrEP initiation was related to PrEP awareness, low income, unstable housing, receipt of navigation services, and reports of noninjection substance use and a recent sexual relationship with an HIV-positive partner [10]. Although some research has been done, there is a need for more focus on health communication with tailoring to the population. Black women shared a limited awareness of PrEP exacerbated by the lack of Black women-specific marketing [9]. In a qualitative study assessing the permanent perceptions Black cisgender women made based on external influences such as media messaging, researchers determined that an internal belief that PrEP is not for heterosexual people was developed because some Black cisgender women felt that commercials and marketing for PrEP were geared toward male same gender–loving couples and people of trans experience [20]. Opportunities to support Black women-specific social marketing could increase awareness and knowledge regarding the potential sexual health benefits of PrEP [9]. Additional research outcomes suggest that Black women want to know that the out-of-pocket costs for PrEP will be affordable, that they will have insurance coverage, that they will have positive interactions with informed and culturally competent clinical staff, access to a discreet and convenient clinic, and that PrEP will be safe for them [10]. Emerging research has demonstrated a growing interest in social media–based sexual health interventions for Black women [21].

### Goal of This Study

This project addressed social and structural determinants of HIV for Black cisgender women by using an intersectional framework that brought together health communication through the lens of culture, race, and gender. Study findings here will contribute to EHE goals by potentially increasing PrEP access among HIV-naïve Black women and informing the Project ROLE intervention, which is currently being developed and will be fully tested to assess the efficacy of a new vlog series among Black cisgender women that will address PrEP knowledge, uptake, persistence, and adherence from the perspectives of both Black women and health care providers of Black women through community health centers and on social media through a NIMH-funded R34 mechanism (1R34MH136826-01). Findings of the Texas Development Center for AIDS Research pilot study substantiated the feasibility of social media as a plausible source of information to Black cisgender women for PrEP access and a pathway to increase uptake for Black cisgender women in Harris County. The purpose of the study was to better understand Black cisgender women’s behaviors when engaging in social media-based HIV prevention interventions designed to provide direct access to web-based sexual health resources related to PrEP. Our research question was, “Can a culturally tailored social media-based vlog HIV prevention intervention motivate Black cisgender women to seek online access to PrEP resources?”

## Methods

### Prior Work

With the awareness of literary findings on the facilitators and barriers to PrEP uptake among Black cisgender women, the research presented here is an extension of a research concept piloted in an emergency department, whereby vlogs, which can be described as videos of blog posts, were used to debunk culturally believed myths in the Black community on how HIV and sexually transmitted infections are transmitted [22-27].

#### Behavioral Framework of the Prior Study

The premise of the health communication message of the vlogs stemmed from findings of a qualitative research study where Black cisgender women enrolled in an emergency department setting described a minimized value for sex [28]. Women who used the word “just” to describe sexual acts demonstrated a higher prevalence of experience with condomless sex, a previous diagnosis of a sexually transmitted disease (STI), and abuse. Misperceptions about what is safe or risky needs to be proven or refuted in order to promote sexual health and inform healthy sexual decision-making. The goal of the vlogs in the prior study that was a response to the research study (2015‐2016) was to resonate with Black women and create an opportunity to replace common misconceptions with the correct information needed to inform healthy sexual decision-making. Based on the protocol of the prior study approved by the University of Texas Health Science Center at Houston–Committee for the Protection of Human Subjects (CPHS), the proposed long-term programming stemming from the pilot study was to explore the utility of social media in reaching marginalized populations.

#### Overview of Methods of the Prior Study

Investigators completed a pilot randomized controlled trial of an HIV prevention intervention of vlogs comparing 2 educational strategies, a storytelling strategy versus an interactive gaming strategy, to assess and compare changes in knowledge pre- and post-intervention. In total, 26 Black cisgender women aged 18‐45 years who reported heterosexual sex within the last 3 months were enrolled [23-27]. The team used actors who reflected the target audience on the premises of race, gender, and colloquialism as the communication method in an effort to enhance cultural relevance with the intervention’s content. The vlog strategy was innovative, relatable, and motivating for Black women.

### Study Design

A test of whether vlogs could motivate online access to PrEP resources among Black cisgender women using 2 aims through a 90-day social media campaign was conducted. The findings of Aim 1, which include the development and testing of the vlog intervention, have been detailed in other studies [10].

During Aim 2, the social media campaign’s performance was evaluated by assessing whether vlog viewership translated to linking Black women in Harris County to local PrEP resources. We assessed the primary outcome, engagement rate by reach, and secondary outcomes, post interactions, and visualizations (ie, views initiated, viewership, and completion rates) and evaluated the social media campaign’s feasibility and acceptability at linking Black cisgender women to local PrEP resources. The social media consultant developed a report with metrics, analytics, and key performance indicators (including engagement with the campaign; vlog reach to Black women, ie, visualization; community engagement; and quantity of vlog material consumed ie, time) that measure study outcomes to quantify how well vlogs performed at motivating independent access to PrEP information as measured by clicks on hyperlinks to local PrEP resources.

### Study Population

The social media campaign aimed to engage adult Black cisgender women aged 18‐45 years who self-reported on their social media page profiles that they were assigned female at birth.

Eligible participants included cisgender women in Harris County who were (1) assigned female sex at birth and currently identified as female, (2) between 18 and 45 years of age, (3) fluent in English, and (4) had a phone or internet access.

### Study Procedures

#### Development of the Marketing Strategy

A certified Social Media Advertisement expert designed a marketing campaign for implementation across social media websites, including Facebook and Instagram, using an algorithm to target individuals self-identifying as female, aged 18‐45 years, usually consume content that is often viewed by Black cisgender women (ie, health and beauty), and reside in neighborhoods (based on zip code) in Harris County where most residents are Black or African American. These women were shown a series of brief vlog snippets on their social media pages, along with a brief message about PrEP and an active hyperlink to local PrEP resources. This strategy included a bi-monthly evaluation with an adjustment approach to reach a balanced sample and sustain active engagement with the target community. The study team reviewed the algorithm and made revisions as necessary before launching the campaign. In 2022, Facebook changed its demographic collection procedures and does not allow researchers to create inclusion criteria and exclude by race. Therefore, it is probable that non-Black women viewed the material.

#### Development of the Study’s Landing Page

The social media ads provided active links to a landing page that housed access to local PrEP resources. The landing page was housed on the academic health center’s website and was co-developed and managed by a web developer and the PI of the grant. The landing page’s content included a link to a brief survey, described as a pre-screening tool, where individuals could describe brief demographic factors (eg, race and gender). Below the link to the brief survey, a question, “Did you Know?” is followed by a few sentences describing the status of HIV in the South with Black women and how the Texas D-CFAR pilot project aims to address the current health inequity. A table titled, “Pre-Exposure Prophylaxis (PrEP) Providers in Houston” is provided with the name of each local PrEP-providing agency, their physical address, and active links to their website and social media pages for ease of access to those who visit the page. The bottom of the landing page contains a link to the Texas-Development CFAR pilot protocol page, which provides more details about the study, inclusive of a project summary, background information, objectives, funding and collaborators, as well as a bio of project leadership [24].

#### Development of 60-Second Vlog Trailers

A study team member with robust vlogging experience on social media created 10 vlog trailers with brief excerpts from each vlog for the social media campaign. The trailers highlighted excerpts on PrEP within engaging aspects of vlogs with intentions to garner the immediate interest of Black women. The study team reviewed all 10 trailers, recommended edits, and collectively chose the top 5. When the trailers were refined and finalized, the social media ad expert launched the campaign with all 5 trailers.

#### Development of Post Content to Accompany Vlog Trailers

Each social media post included written content with at least 280 characters, including the hyperlink to local PrEP resources on the study’s landing page. An example of a post was, “What does it mean to be at risk for HIV? Check out the vlog and click on the link to learn more about PrEP” (83 characters). We included hashtags like #PrEPforHER, #OWNYourHealth, and #PreventHIV, when character limits allowed.

#### Launching the Campaign

The social media ad expert launched the campaign on March 1, 2023, and concluded on September 8, 2023. Viewers of the vlog trailers were identified by an algorithm led by the social media ad expert who mobilized clicks on advertisements through social media sites using a strategy with established effectiveness. The social media ad expert tracked whether viewers clicked on referral sources. His algorithm determined how often the trailers were shown to social media audiences. Through careful monitoring on a weekly basis, the trailers with the least activity were removed from the campaign in an iterative fashion. The highest performing trailers (n=2) remained for the duration of the campaign. There was no participant enrollment process for individuals to view the social media campaign, and only publicly available data associated with paid advertisements were used to collect data. Individuals were not engaged on an individual basis. The social media campaign was publicly available.

#### Access to Local PrEP Clinics Through the Social Media Campaign

The project links cisgender women in an EHE priority jurisdiction to local PrEP resources, providing them with access to local PrEP clinic information through a landing page from a social media campaign, and introduced PrEP as an effective HIV prevention option through highly trafficked social media channels as a means of encouraging PrEP initiation with Black cisgender women in Harris County using vlogging for sexual health. Harris County residents who viewed the vlogs on their social media page had access to more information about PrEP by clicking on hyperlinks to local PrEP resources that accompany the vlog trailers. Hyperlinks were connected to websites and social media pages of local PrEP providers for review and following.

### Ethical Considerations

The University of Texas Health Science Center at Houston–CPHS determined this study did not meet the regulatory definition of human subjects’ research. Thus, no further review was required.

### Data Analysis

A report developed with Facebook Insights and Google Analytics was used to evaluate the performance of the social media campaign. The analytic strategy collected data based on age, gender, race, and interest in health and beauty. Descriptive statistics assessed the self-identified gender and age of the profiles of individuals who engaged with the campaign. The study team also evaluated key performance indicators that included impressions (the number of times a user saw the vlog), video plays (the number of times a video starts playing), and the number of clicks to local PrEP resources (the total number of times users have clicked on a specific link). The study team also evaluated the economic cost of implementing a sexual health–focused social media campaign to increase PrEP access to Black cisgender women.

## Results

### Intervention Characteristics

Social media platforms that were leveraged for the campaign included Facebook and Instagram. Key performance indicators of the social media campaign stratified by age revealed that young people aged 18‐24 years engaged most with the campaign (Table 1). Video plays at 50% of the vlogs (n=30,877) were most common among women aged 18‐24 years (n=12,017) and least common among women aged 45‐54 years (n=658). Video plays at 50% means that individuals started playing the video and watched 50% of the video. Table 2 shows an overview of women engaging with the social media campaign by age. Across cohorts, women aged 18‐24 years were mostly represented, followed by a decrease in engagement with social media campaign in each subsequent cohort, with women aged 45‐54 years being the least represented.

**Table 1.** Key performance indicators of the social media campaign, stratified by age (March-September 2023).

Age (years)	Link clicks	Reach	Impressions	Cost per link click (USD)	Amount spent (USD)	Video plays at 25%	Video plays at 50%	Video plays at 75%	Video plays at 95%	Video plays at 100%	Video plays
18‐24	2330	39,968	491,656	$1.27	$2967.69	15,752	12,017	10,192	7072	6413	465,262
25‐34	1834	38,816	336,524	$1.27	$2337.52	16,741	10,129	7494	5652	4913	302,026
35‐44	1662	29,376	250,782	$1.28	$2124.98	14,534	8073	5204	4292	3567	217,758
45‐54	126	2592	19,667	$1.43	$180.76	1255	658	413	335	286	17,198
Total	5952	110,752	1,098,629	$1.28	$7610.94	48,282	30,877	23,303	17,351	15,179	1,002,244

**Table 2.** Age distribution of women engaging with the social media campaign over 6 months.

Age group (years)	Total count (approximate)
13-17	0
18-24	2250
25-34	1800
35-44	1600
45-54	100
55-64	0
65+	0

**Figure 1.**
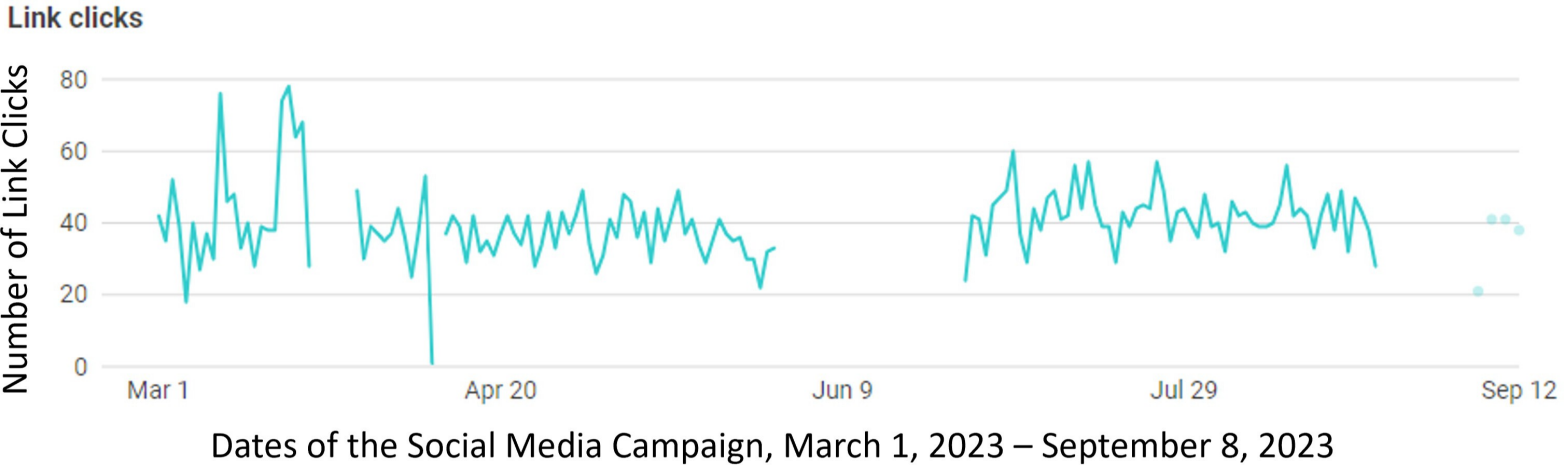
Summary of the trend in link clicks over the breadth of the social media campaign.

A line graph was used to assess the trend in clicks of the campaign over time (Figure 1). The trend reveals a steady link click rate of around 40 across the breadth of the 6-month campaign, largely within a 20‐60 link click rate.

**Figure 2.**
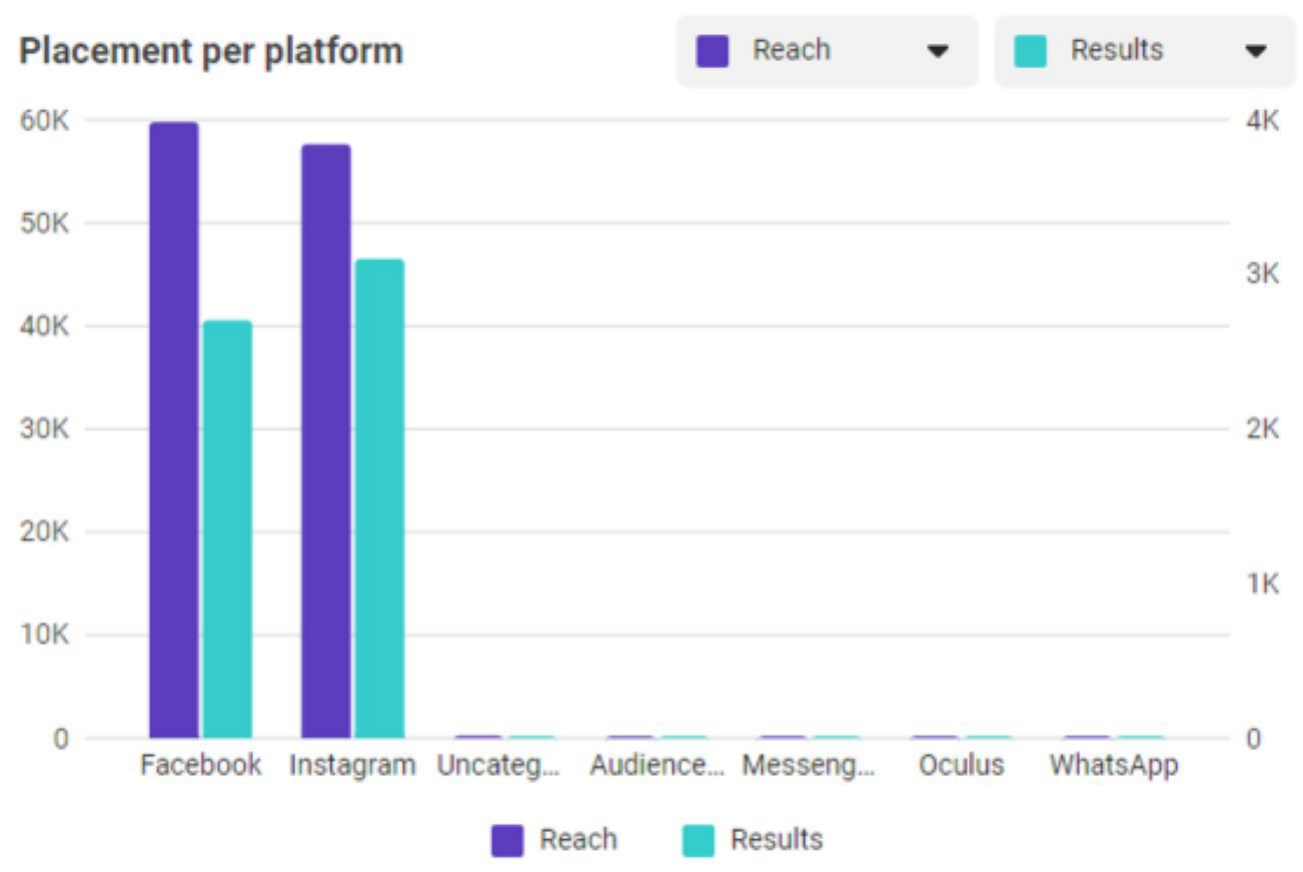
Social media campaign performance based on placement per platform with a mobile device only. All platforms apart from Facebook and Instagram have a value of 0.

The final report showed 1,098,629 impressions and 1,002,244 total video plays and resulted in 15,952 link clicks to local PrEP resources. The campaign reached more individuals on Instagram (59,776) than on Facebook (57,623), but there were more clicks on Facebook (3101) than on Instagram (2703) (Figure 2).

The evaluation of the economic cost of the social media campaign revealed that the cost was $1.28 per link click. The total amount spent was $7410.94 across the 6-month campaign. When stratified by social media platform, the campaign performed slightly better on Instagram than on Facebook, costing $3992.85 versus $3411.55, respectively (Table 3).

**Table 3.** Key performance indicators: by overall campaign and by platform (March-September 2023).

Key performance indicators and measures		Value
Overall social media campaign		
Reach (post engagement), n		110,752
Total impressions, n		1,098,629
Link clicks, n		5952
Cost (USD)		$7410.94
Cost per click (USD)		$1.28
Platform		
Facebook		
Reach, n		57,623
Link clicks, n		3101
Cost (USD)		$3411.55
Instagram		
Reach, n		59,776
Link clicks, n		2703
Cost (USD)		$3,992.85

## Discussion

### Study Findings and Comparison With Previous Works

The social media campaign over 6 months reached close to 6000 women. The campaign primarily used Facebook and Instagram. The highest engagement was observed among individuals aged 18‐24 years, particularly women, who accounted for the most video plays (n=12,017). Engagement was the lowest among women aged 45‐54 years (n=658). The distribution of engagement showed a consistent decline in participation across older age groups, with women aged 18‐24 years being the most represented and women aged 45‐54 years the least. The final report indicated 1,098,629 impressions and 1,002,244 total video plays, leading to 15,952 link clicks to local PrEP resources. The campaign reached more individuals on Instagram (59,776) than on Facebook (57,623), but Facebook generated more clicks (3101) compared to Instagram (2703).

When exploring the distribution of women who engaged with the social media campaign, it was evident that younger women were more likely to be represented. This finding supports existing research demonstrating that mobile health apps or electronic sexual and reproductive health interventions are effective in engaging young Black women [29,30]. Additionally, the findings align with previous research demonstrating that generational differences exist in the ways in which women access health information [28]. Many people access health information through their smartphones as the first step in gathering valid and correct information. This may be different from older generations, such as older millennials, generation X, or boomers, who developed sexual and reproductive health practices before the invention and proliferation of the internet and, as such, sought out professionals such as physicians, nurses, and other health care providers as trusted sources of sexual and reproductive health information. While seeking out medical professionals is a trusted and effective route, mobile health and e-health apps now remove the barrier of access to and utilization of health care and providers, due to the nature of needing insurance or being insured, in addition to costs related to co-pays, transportation, and time to take off work [29]. Additionally, these approaches to HIV prevention remove the burden of actively participating in one’s sexual health wellness and accommodate diverse lifestyles [29,31]. Moreover, the findings of this study demonstrate that women aged 18‐35 years engaged in the social media campaign. Given that newer cases of STIs such as chlamydia, gonorrhea, and syphilis are the highest among women aged 18‐25 years [19] and new HIV diagnoses are reported among women aged 25‐35 years [19], it is advantageous to explore the sustainability and scale-up of such an intervention to address the current public health needs. The decrease in engagement as age increased could indicate the need for tailored approaches in future campaigns, perhaps targeting specific age groups with content that resonates more with their interests and preferences [32].

Silva et al [29] sampled male and female youth aged 16‐49 years on social media (including Twitter, subsequently rebranded X, and Facebook), along with other mass media platforms, in a study supporting intergenerational communication on sexual reproductive health and family planning with youth. While findings show strong potential, findings also showed that young people still need supportive guidance and approval from trusted adults in their region of the world (eg, sub-Saharan Africa) [29]. Another study assessed sexual health promotion campaigns on Twitter and Facebook and found significant potential with health promotion initiatives based on the number of interactions with users [29]. Although social media has been explored for health promotion and sexual health generally, the focus on PrEP engagement appears to be missing. This reveals a meaningful gap and unmet need that this study was poised to fill.

Although minimal, a greater number of women used Instagram as opposed to Facebook. However, link clicks on Facebook were higher than that on Instagram. It would be advantageous to retain both social media sites for future programming, as each site tends to garner a different viewership demographic [33]. Despite the differences in user preferences, both platforms were deemed acceptable to women engaged in the social media campaign over 6 months.

Compared to the over 1 million impressions and over 1 million total video plays, clicks to the local PrEP resources were close to 16,000, demonstrating that while women are interested in the campaign and videos, there is less engagement with PrEP. This may point to a critical point in women’s decision-making in terms of their self-assessment of HIV risk or vulnerability and the need to try a new HIV prevention strategy. The novelty of the innovation reinforces the fact that such social media campaigns for Black women are scant, and the scale-up of these interventions would make engaging in HIV prevention practices commonplace and more widely adopted. This study, however, is the first of its kind to actually embed local PrEP resources within the vlogs to drive traffic to the clinics. This is an ideal strategy as it addresses the retention of participants. Findings from this study suggest that social media campaigns as an intervention strategy to increase PrEP access are likely a compatible complement to EHE goals and outreach efforts to enhance access to PrEP providers among populations who are vulnerable to new HIV diagnoses. Video plays were over 1 million views, surpassing the expectations of the investigative team while solidifying the feasibility of this approach at reaching cisgender women through social media with PrEP messaging.

### Limitations

There are limitations to our study. First, there were 2 breaks in the campaign, stalled by communication gaps within the team and logistical challenges within the institution occurring between March 23, 2023 and March 30, 2023 and May 30, 2023 and June 27, 2023. Although this occurred, our findings remain robust, and if the study team had not had breaks in the campaign, the results could have been similar or higher for reach, clicks, vlog views, and accessing local PrEP clinic resources. Second, in regard to the racial profile of the study population, the ability of any social media campaign on Facebook or Instagram to market advertisements by race was removed. In lieu of this sudden and unpredicted change, the study team and consultant advertisement expert used 2 proxies in lieu of race, which were content, specifically health and beauty, and zip codes in areas where a significant majority were Black. As an example, zip code 77033 was one of several zip codes used. According to the United States Postal Service, the majority of residents in 77033, specifically 75.4% as of January 13, 2025, are Black or African American. Individuals who resided in zip code 77033 often viewed health and beauty content and described themselves as female; they were shown the vlogs during the social media campaign. Based on the expertise of our consultant, the team was confident that this approach would garner viewership from a majority Black female population. However, it is likely that the vlogs were viewed by a minority of non-Black women.

### Future Research

Future studies examining implementation outcomes such as acceptability could engage the users in focus groups or in-depth qualitative interviews to determine reasons for staying on the site or linking to local PrEP clinics. Given that many clinics operate on limited budgets, it may be advantageous to invest in a formal feedback session on their webpage for opportunities to garner usage of the site further and enhance the user experience and ability to get the information needed quickly. Future studies could also investigate the appropriateness of such an intervention. Additionally, the utilization of a hybrid type 2 design to examine the scale-up and testing of both the intervention effectiveness and implementation strategy would allow for the simultaneous assessment of HIV prevention via vlogs with multiple sites.

### Conclusions

The campaign demonstrated the feasibility, acceptability, and appropriateness of this approach with Black cisgender women and illustrated preliminary effectiveness at supporting access to local PrEP resources with Black cisgender women. Further dissemination and implementation of this approach is necessary to fully assess whether vlog viewership and clicks on links to PrEP resources can meaningfully empower Black cisgender women to access PrEP and help them to assess whether PrEP is personally a useful HIV prevention option.

## Abbreviations

CPHS: University of Texas Health Science Center at Houston’s Committee for the Protection of Human Subjects
EBI: evidence-based intervention
EHE: ending the HIV epidemic
PrEP: pre-exposure prophylaxis
STI: sexually transmitted disease
